# Cardiac MRI Based Left Ventricular Global Function Index: Association with Disease Severity in Patients with ICD for Secondary Prevention

**DOI:** 10.3390/jcm10214980

**Published:** 2021-10-27

**Authors:** Andreas Leonhard Schober, Carsten Jungbauer, Florian Poschenrieder, Alexander Daniel Schober, Ute Hubauer, Andreas Keyser, Sabine Fredersdorf-Hahn, Kurt Debl, Lars S. Maier, Samuel Sossalla, Stefan Buchner, Ekrem Üçer

**Affiliations:** 1 Klinik und Poliklinik für Innere Medizin II, Universitätsklinikum Regensburg, 93053 Regensburg, Germany; carsten.jungbauer@ukr.de (C.J.); alexander.schober@ukr.de (A.D.S.); ute.hubauer@ukr.de (U.H.); sabine.fredersdorf@ukr.de (S.F.-H.); kurt.debl@ukr.de (K.D.); lars.maier@ukr.de (L.S.M.); samuel.sossalla@ukr.de (S.S.); ekrem.uecer@ukr.de (E.Ü.); 2 Institut für Röntgendiagnostik, Universitätsklinikum Regensburg, 93053 Regensburg, Germany; florian.poschenrider@ukr.de; 3 Klinik und Poliklinik für Herz-, Thorax- und Herznahe Gefäßchirurgie, Universitätsklinikum Regensburg, 93053 Regensburg, Germany; andreas.keyser@ukr.de; 4 Innere Medizin II, Sana Kliniken des Landkreises Cham, 93413 Cham, Germany; stefan.buchner@sana.de

**Keywords:** ICD, heart failure, cardiac arrest, cardiac magnetic resonance, ejection

## Abstract

Left ventricular (LV) ejection fraction (LVEF) is the most widely used prognostic marker in cardiovascular diseases. LV global function index (LVGFI) is a novel marker which incorporates the total LV structure in the assessment of LV cardiac performance. We evaluated the prognostic significance of LVGFI, measured by cardiovascular magnetic resonance (CMR), in predicting mortality and ICD therapies in a real-world (ICD) population with secondary ICD prevention indication, to detect a high-risk group among these patients. In total, 105 patients with cardiac MRI prior to the ICD implantation were included (mean age 56 ± 16 years old; 76% male). Using the MRI data for each patient LVGFI was determined and a cut-off for the LVGFI value was calculated. Patients were followed up every four to six months in our or clinics in proximity. Data on the occurrence of heart failure symptoms and or mortality, as well as device therapies and other vital parameters, were collected. Follow up duration was 37 months in median. The mean LVGFI was 24.5%, the cut off value for LVGFI 13.5%. According to the LVGFI Index patient were divided into 2 groups, 86 patients in the group with the higher LVGFI und 19 patients in the lower group. The LVGFI correlates significantly with the LVEF (*r* = 0.642, *p* < 0.001). In Kaplan–Meier analysis, a lower LVGFI (<13.5%) was associated with a higher rate of mortality and rehospitalization (*p* = 0.002). In contrast, echocardiographic LVEF ≤ 33% was not associated with a higher rate of mortality or rehospitalization. Multivariate Cox-regression analysis revealed a lower LVGFI (*p* = 0.025, HR = 0.941; 95%-CI 0.89–0.99) and diabetes mellitus (*p* = 0.027, HR = 0.33; 95%-CI 0.13–0.88) as an independent predictor for mortality and rehospitalization. There was no association between the combined endpoint and the LVEF_MRT_, LVEF_echo_, NYHA > I, the initial device or a medication (each *p* = n.s.). Further, in Kaplan–Meier analysis no association was evident between the LVGFI and adequate ICD therapy (*p* = n.s.). In secondary prevention ICD patients reduced LVGFI was shown as an independent predictor for mortality and rehospitalization, but not for ICD therapies. We were able to identify a high-risk collective among these patients, but further investigation is needed to evaluate LVGFI compared to ejection fraction, especially in patients with an elevated risk for adverse cardiac events.

## 1. Introduction

Left ventricular (LV) ejection fraction (LVEF) is the most widely used parameter in risk stratification in patients with cardiovascular disease [1]. Furthermore, in patients without a previous life-threatening ventricular arrhythmia, the decision to implant an implantable cardioverter defibrillator (ICD) is based on the LVEF measure [2]. In patients who received their ICD in secondary prevention, the LV dysfunction is still an important prognostic marker [3,4,5]. Nevertheless, the sensitivity and specificity of LVEF is being questioned in recent years since in some patients with ICD appropriate therapies never occur despite low LVEF (≤35%) and others with LVEF greater than 35% do receive appropriate therapies [2]. Therefore, new prognostic markers for patients with cardiac coronary vascular diseases have been developed in recent years, which incorporate structural changes in the LV myocardium, such as hypertrophy, myocardial mass and similar parameters. Such an emerging marker is the LV global function index (LVGFI).

In prior studies strong correlations had been shown between LVGFI and severe myocardial and microvascular damage in patients after myocardial infarction, as well as a high predictive value of LVGFI in predicting major cardiac adverse effects [6,7]. In our study we explored the value of LVGFI in predicting mortality and appropriate ICD therapies in patients who received an ICD, after surviving a cardiac arrest, to identify a high-risk collective in secondary prevention ICD patients who are missing good prognostic markers at the moment.

## 2. Materials and Methods

### 2.1. Study Design

**Figure 1:**
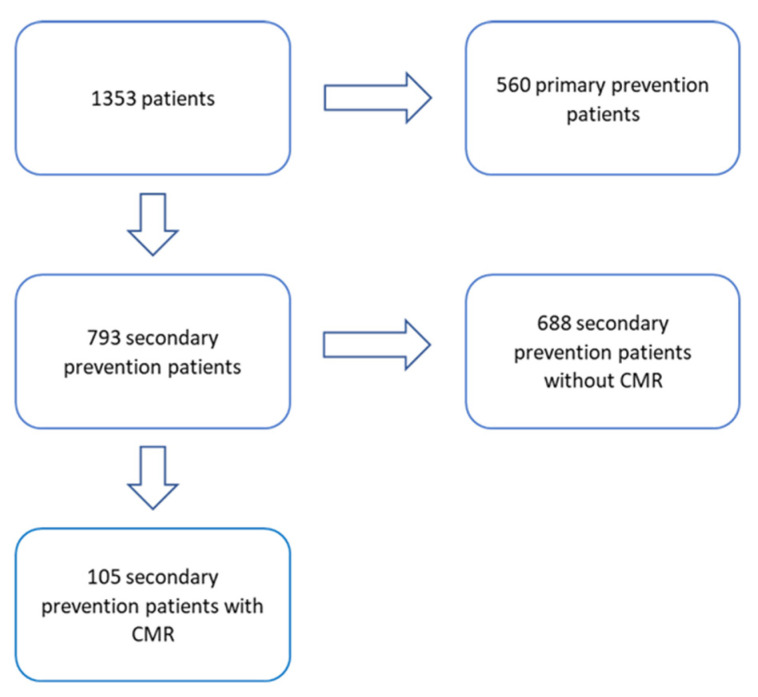
Flowchart showing the distribution of the Regensburger ICD-survival trial (Res-IST) patients divided by the sort of prevention and the presence of a cardiac magnetic resonance imaging (CMR).

The study cohort consisted of patients enrolled in a prospective observational study of the ICD register at the University Hospital Regensburg. All patients referred to our department for ICD implantation between 1992 and 2018 with ICD implantation for secondary prevention were recruited into the study. Those without MRI before the ICD implantation were excluded, thus one-hundred-five patients with ICD indication for secondary prevention, in whom cardiac magnetic resonance imaging (CMR) was performed before the ICD implantation were included (in Figure 1).

Clinical follow-up and device interrogations of all the patients had been performed in our clinic or in clinics in our proximity, from where all the examination data could be obtained. Device readouts were scheduled on a quarterly basis. Exclusion criteria were age below 18 years and unwillingness to sign the informed consent form.

We included all the device readouts, as well as clinical follow ups, including MRI data, into an electronic database.

The local Ethics Committee approved the study protocol based on the regulations stated in the Helsinki Declaration of Good Clinical Practice. Prior to enrolment, written informed consent was obtained from each participant.

### 2.2. Study Endpoints and Follow-Up

The primary endpoints of this study were all-cause mortality and rehospitalization due to heart failure. Rehospitalization due to heart failure was defined as readmission to hospital for management of heart failure (defined by the presence of new symptoms of paroxysmal nocturnal dyspnea, orthopnea, or edema with one or more concurrent signs, including ventricular gallop rhythm, jugular venous distention, bilateral rales in at least the lower third of the lung fields, elevated venous pressure, or pulmonary venous congestion on chest X-ray with interstitial or alveolar edema).

Secondary endpoints were appropriate ICD therapies and were defined as an episode of ventricular tachycardia or ventricular fibrillation treated with anti-tachycardia pacing (ATP) or device shock.

### 2.3. Cardiac Magnetic Resonance

In all patients a contrast enhanced CMR had been performed before the ICD implantation. CMR scans were performed on a 1.5-T AVANTO-scanner (Siemens, Erlangen, Germany) according to a standardized protocol. Evaluation of images was performed manually using standard software (ARGUS, Siemens, Erlangen, Germany). In brief, cine images in short axis were acquired using breath-hold, retrospective electrocardiogram triggered true fast imaging with steady-state free precession (FISP) bright blood sequences. The LVGFI was determined according to the formula published by Mewton et al. [7].

LVGFI (%) = LVSV (mL)/LVGV (mL) × 100;

where LVSV is the left ventricular stroke volume and LVGV is the left ventricular global volume. The left ventricular global volume was calculated according to the following formula:

LVGV (mL) = {(LVEDV (mL) + LVESV (mL))/2} + Volume LV myocardium (excluding trabeculaes and the papillary muscles)

where LVEDV is the left ventricular end-diastolic volume, and LVESV the left ventricular end-systolic volume. The presence of late gadolinium enhancement (LGE) was detected, but its quantification is not included in our regular CMR protocol.

### 2.4. Defining Etiology

Coronary angiography was performed in 104 cases (one patient had the diagnosis of Hypertrophic Cardiomyopathy (HCM) since the age of 13 and received the ICD at the age of 19 without a coronary an-giography before implantation), the diagnosis of the underlying cardiac disease based on a combination of 12-lead ecg, lab work, exercise testing, echocardiography, coronary angiography and the MRI as recommended by the guidelines [8].

### 2.5. Device Readout

Each patient was followed up in our institution at 4–6 weeks after the implantation; thereafter every 3–6 months. During each visit device related parameter including lead function, appropriate and inappropriate therapies, device related complications, battery status, device history, and device programming were evaluated and documented. If patients were followed elsewhere than in our institution the data were requested from the corresponding institution.

### 2.6. Defining the Device Therapies

Appropriate therapy was defined as an episode of ventricular tachycardia or ventricular fibrillation treated with anti-tachycardia pacing (ATP) and shock. Inappropriate therapies were ATP or shock therapies in the case of a supraventricular arrhythmia (atrial fibrillation, atrial flutter, Atrioventricular nodal reentry tachycardia (AVNRT), Atrioventricular reentrant tachycardia (AVRT), sinus tachycardia, focal atrial tachycardia) or an oversensing due to lead issues or due to electromagnetic interferences. If an episode was treated with both ATP and shock, shock therapy was selected as the endpoint.

### 2.7. ICD Programming

The ICDs were programmed according to our institutional standards with one therapeutic ventricular tachycardia (VT)-zone and one ventricular fibrillation (VF)-zone as follows: up to 4 sequences of ATP in patients with marked LV dysfunction (ejection fraction < 40%) and 6 sequences of ATP in patients with slightly reduced and normal LV function (ejection fraction > 40%). Sustained rate duration was deactivated in all patients.

The first ATP sequence was a scan train (Boston and SJM: 88% coupling interval 88% burst cycle length, 10 ms decrement; Medtronic: 88% coupling interval, Z 10 ms decrement; Biotronik: 90% coupling interval, 10 ms decrement) and the second sequence was a ramp train (Boston, SJM: 88% coupling interval, 84% burst cycle length, 10 ms decrement; Medtronic: 91% coupling interval; Biotronik: 85% coupling interval, 10 ms S1-decrement).

In those with documented VT above 200 bpm the VT zone was programmed 10 to 15 bpm slower than the slowest documented VT rate. If the VT was hemodynamically tolerable, we programmed only ATP therapies; but if not tolerable, both ATP and shock therapy were programmed. The VF zone was programmed at a rate above 240 bpm consisting of 1 ATP during charging (if available) and shock therapies.

In inherited arrhythmia syndromes such as Brugada Syndrome, long QT, and short QT syndrome we programmed a monitor zone from 187 to 220 bpm with a detection time of 60 s and a VF zone starting from 220 bpm with 5 s detection time as the expected arrhythmia is a ventricular fibrillation. If there was no possibility to program a detection time, 60 intervals were chosen for the VT zone and 20 for the VF zone.

### 2.8. Statistical Analysis

Continuous, normally distributed variables (Shapiro–Wilk test) are expressed as mean ± standard deviation, not normally distributed continuous variables as median with interquartile range. Categorical variables are displayed as the number and percentage. Mann–Whitney-*U* test was used to determine differences in continuous variables between groups. Pearson or Spearman r correlations were calculated as appropriate.

Outcome functions were estimated using Kaplan–Meier analysis and groups were compared using the log-rank test. Univariate and multivariate Cox regression analysis was applied to identify parameters associated with outcome. Only variables with a *p* < 0.05 in univariate analysis were entered in multivariate models. The predictive value of LVGFI and of other CMR parameters, as well as of EF determined with echocardiography was assessed using receiving operators’ curve (ROC) analyses. After determining the specificity and sensitivity values from the ROC analysis we calculated the Youden index for LVGFI, LVEF_MRI_, and LVEF_echo_ with the best predictive value. These values were then used as dichotomized categorical variables for outcome analysis, as well as categorizing the patient population.

The Youden index (J) was calculated using the following formula:

J = sensitivity + specificity − 1

All statistical tests were two-tailed, and *p* < 0.05 was considered statistically significant. Statistical analysis was performed with SPSS 22.0 (SPSS Inc., Chicago, IL, USA).

## 3. Results

### 3.1. Study Population

Baseline characteristics of the 105 patients (mean age 56 ± 16 years; 76% male) are shown in Table 1. In total, 41% of patients had a coronary artery disease, 25% of the study population a previous myocardial infarction, and 37% had a cardiomyopathy. Overall, 39 patients suffered from a cardiomyopathy: 20 cases with dilated cardiomyopathy, 5 cases with HCM or hypertrophic obstructive cardiomyopathy (HOCM), 3 cases with arrhythmogenic right ventricular dysplasia (ARVD), in 4 cases with Tako Tsubo cardiomyopathy, 6 cases with residuals after myocarditis, and in 1 case from non-compaction. In total, 9.5% of the patients had a primary VF without any detectable structural heart disease and 4.8% idiopathic VT; 7.6% had a long QT-Syndrome. The cause of cardiac arrest were monomorphic VTs in 43.8%, polymorphic VTs in 4.8%, and fast VTs in 1.0% of patients. VF occurred in 45.6% of the cases. In 4.8% of the cases an asystole was detected as initial rhythm (Table 1). According to the underlying heart disease and LV function, single-chamber ICD was implanted in 59%, dual-chamber ICD in 23.8%, implantable cardiac resynchronization therapy defibrillators (CRT-D) in 6.7% and subcutaneous implantable cardioverter defibrillators (S-ICD) in 10.5%. The baseline characteristics such as age, sex, body weight, blood pressure was similarly distributed between both groups. Medical therapies at baseline and basic risk factors were not different between the two groups.

### 3.2. Calculation of the Best Predictive Value for LVGFI, LVEF_MRI_, and LVEFe_cho_

According to the ROC analysis regarding the combined outcome events, the Youden index for LVGFI, LVEF_MRI_, and LVEF_echo_ had been calculated. These were 13.5% for LVGFI, 40.5% for LVEF_MRI_, and 33% for LVEF_echo_. The LVGFI correlated significantly and positively with the LVEF_echo_ (*r* = 0.726, *p* > 0.001) and the LVEF_MRI_ (*r* = 0.944, *p* > 0.001).

### 3.3. Classifying the Groups According to the LVGFI

Using the Youden index we calculated an LVGFI value as the discriminator for the prognosis; according to that, an LVGFI value of above 13.5% was the threshold for the LVGFI differentiating patients with better or worse prognosis (patients with LVGFI ≤ 13.5% having a worse prognosis). Table 2 shows the classification of the patients according to the LVGFI value. A total of 19 patients (18%) had an LVGFI ≤ 13.5% and 86 (82%) an LVGFI > 13.5%. The mean LVGFI was 10 ± 2.5% in the lower LVGFI group vs. 28 ± 10% (*p* < 0.001) in the higher LVGFI group. In the group with LVGFI ≤13.5% significantly more patients (68% vs. 14%, *p* < 0.001) had an EF ≤ 35%. LV function in these patients was significantly lower independently of the technique used to measure the EF, either with echocardiography or with MRI. Similarly, patients with lower LVGFI clinically more often suffered from heart failure and more often had severe symptoms (NYHA class ≥ II). Diabetes was more commonly seen in patients with lower LVGFI. Usage of diuretics, spironolactone and amiodarone/sotalol was significantly higher in patients with lower LVGFI. LGE was detected in 68 cases (13 (68.4%) in the lower group vs. 55 (80.1%) in the higher group, *p* = not significant (n.s.)) (Table 2).

### 3.4. Combined Endpoint: All-Cause Mortality and Hospitalization Due to Heart Failure

**Figure 2:**
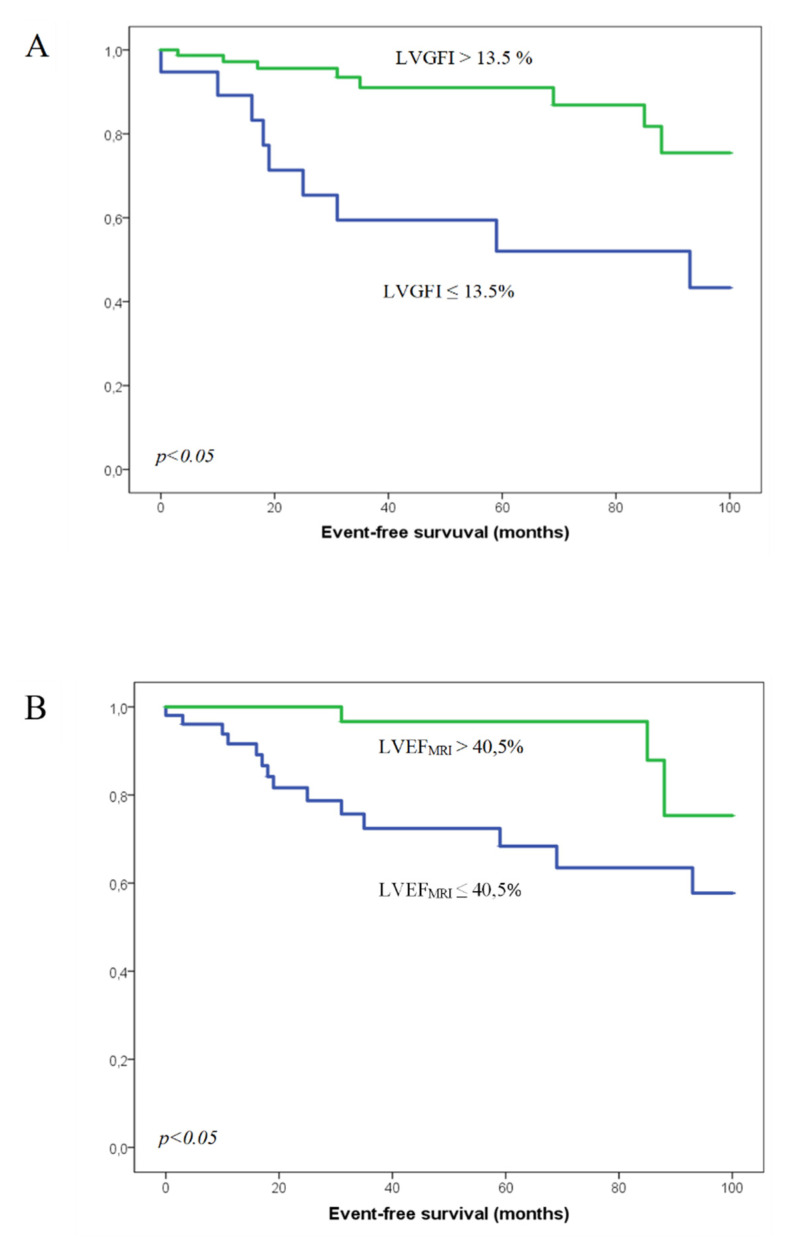

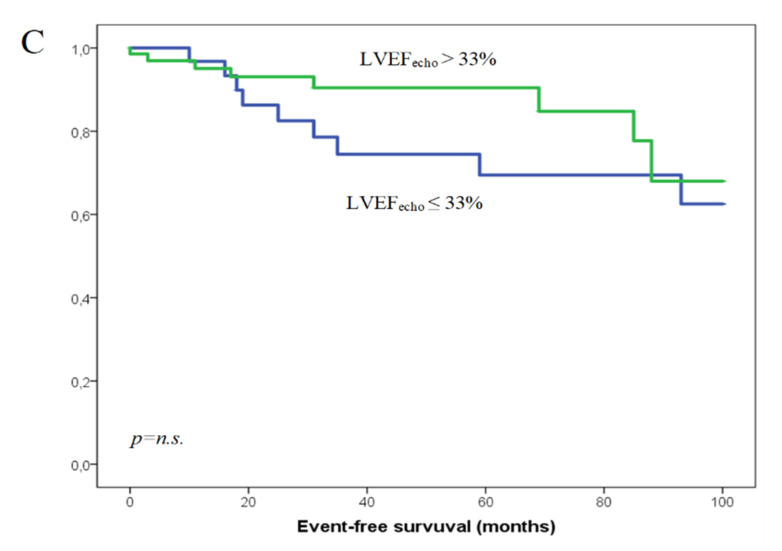
Groups divided by LVGFI (**A**), LVEF measured by MRI (**B**) and LVEF measured by echocardiography (**C**). The green line represents the group with the LVGFI or LVEF above the cut-off value, the blue line the group with the lower LVGFI/LVEF; n.s.: not significant.

The patients were followed up for 37 months in median (IQR 13–84 months). In total, 17 out of 105 patients had reached the endpoint consisting of death or hospitalization due to heart failure (16%). Of these, 9 (47%) out of 17 patients with events were in the lower LVGFI group vs. 8 (9%) in the higher LVGFI group. The patients who reached the combined endpoint showed a significantly lower LVGFI, LVEF_echo_, and LVEF_MRI_ than event-free patients (LVGFI 13.3 ± 10.1%, LVEF_MRI_ 30.5 ± 19.1%, LVEF_echo_ 36.8 ± 17.1% for patients with event, LVGFI 27.4 ± 12.0%, LVEF_MRI_ 44.3 ± 17.1%, LVEF_echo_ 45.5 ± 13.6% for event-free patients) (Figure 2A–C).

### 3.5. Appropriate ICD Therapies

**Figure 3:**
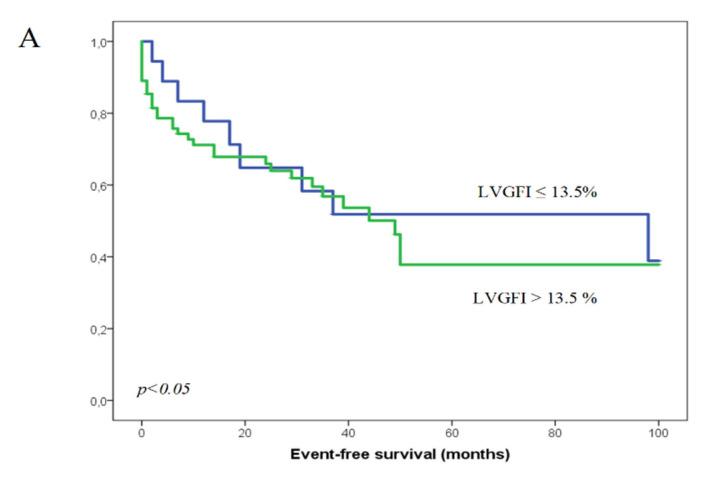

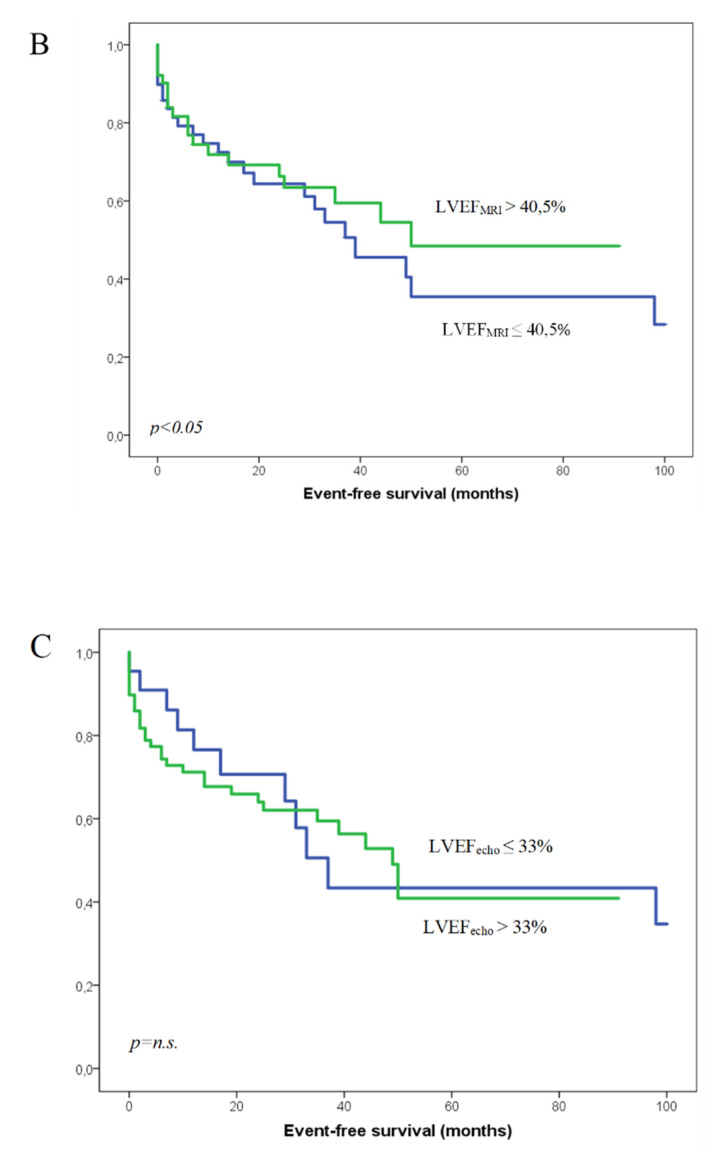
Appropriate ICD-therapies. Groups divided by LVGFI (**A**), LVEF measured by MRI (**B**) and LVEF measured by echocardiography (**C**). The green line represents the group with the LVGFI or LVEF above the cut-off value, the blue line the group with the lower LVG-FI/LVEF.

In 5 patients we could not retrieve data about the device therapy. In total, 43 (43%) of 100 patients had appropriate device therapies; out of those, 9 (50%) of 18 patients were in the lower LVGFI group and 34 (41%) of 82 in the higher LVGFI group. There were no significant differences in LVGFI, LVEF_echo_, and LVEF_MRI_ in patients with and without appropriate therapies (Figure 3A–C). Out of those 43 patients 24 received only one appropriate therapy during follow up, 19 received multiple appropriate therapies. VT ablation was performed in 6 cases. There was no significant difference in the occurrence of multiple appropriate therapies or the performance of VT ablations between patients with a higher LVGFI and a lower LVGFI (17 patients with multiple appropriate therapies in the greater group vs. 7 in the lower one, *p* = n.s; 4 VT ablations in the greater group vs. 2 in the lower group *p* = n.s.).

### 3.6. Inappropriate ICD Therapies

In total, 20 inappropriate therapies were detected (7 in the lower group vs. 13 in the higher group), 9 including shock therapies. In Kaplan–Meier analysis (not shown) the LVGFI did not prove to be able to predict the occurrence of inappropriate therapies (*p* = n.s.).

### 3.7. Performance of LVGFI in Predicting the Outcome

Kaplan–Meier analysis showed higher event rate in patients with lower LVGFI (≤13.5%) and LVEF_MRI_. Patients with an LVGFI ≤ 13.5% had significantly worse prognosis than in patients with higher LVGFI (*p* = 0.002). Similarly, patients with LVEF_MRI_ ≤ 40.5% had more events such as hospitalization or death (*p* = 0.009). Conversely, LVEF_echo_ with a cut-off value of 33% did not discriminate patients with events from those without (*p* = 0.09) (Figure 2A–C). Similarly, neither of these values could differentiate patients with appropriate device therapies from those without (*p* = 0.69 for LVGFI, *p* = 0.51 for LVEF_MRI_, *p* = 0.77 for LVEF_echo_) (Figure 3A–C).

### 3.8. Independent Predictors for Worse Outcome

LVGFI, LVEF_MRI_, LVEF_echo_, presence of diabetes, NYHA ≥ II, diuretic usage, spironolactone usage, amiodarone/sotalol usage and female sex were parameters with significant differences between patients with lower and higher LVGFI (Table 2). We also added age at the time of implantation to the analysis as age is a well-known risk factor for all-cause mortality. We included these parameters into a Cox-regression analysis which revealed two independent predictors of worse prognosis, which were a reduced LVGFI (HR = 0.938; 95%-CI 0.887–0.991, *p* = 0.023) and age at implantation (HR = 1.06; 95%-CI 1.014–1.107, *p* = 0.11). The LVEF_MRI_, LVEF_echo_, presence of diabetes, NYHA ≥II, diuretic usage, spironolactone usage, amiodarone/sotalol usage and female sex were, in contrast, no independent predictors for the occurrence of events (Table 3).

## 4. Discussion

Our findings demonstrate, for the first time in a secondary ICD population, that LVGFI is associated with long term outcome. It is a independent predictor of all-cause mortality and hospitalization for heart failure. Although patients with previous cardiac arrest have a high mortality risk, the LVGFI can further stratify patients into higher and lower risk groups.

Our data mean that LV structural components of cardiac remodeling have important effects on the outcome, also in secondary prevention ICD patients and may be able to characterize a collective among these patients who need a closer clinical follow up.

There were several studies that showed a predictive value of the LVGFI, but none of them featured ICD-patients. It was already shown that LVGFI had more prognostic value than LVEF_echo_ in different patient populations. Mewton et al. investigated 5004 patients with atherosclerosis and found out that LVGFI was a significant independent predictor for all cardiovascular events such as heart failure, coronary events such as myocardial infarction cardiac arrest and death from coronary disease [7]. Later, Eitel et al. and Pezel et al. assessed the predictive value of LVGFI in cardiovascular events after ST-elevation myocardial infarction [9,10]. They showed that LVGFI strongly correlates with markers of severe myocardial and microvascular damage. According to their data, the LVGFI had incremental prognostic value in addition to LVEF for prediction of all-cause mortality. Furthermore Huang et al. were able to show that the LVGFI is superior to the LVEF in predicting adverse events in patients with a cardiac amyloidosis [11].

Compared to the measurements of Aquaro et al. in a group of healthy subjects the patients in our study had a much lower LVFGI, as expected. Aquaro et al. found a mean LVGFI of 45% in a patient population of 51 to 60 years old patients [12]. This finding is in contrast with our patient population as all our patients had structural heart disease. Similarly, Reinstadler et al. showed lower LVGFIs in patients with myocardial infarction and also found out that the low LVGFI correlated with adverse events [6].

In their study, Nwabou et al. included 4107 participants (mean age 29.8 years) and followed them for 25 years. Primary endpoints were development of cardiovascular disease and heart failure. In the follow up period 207 of the 4107 participants had reached a primary endpoint. Interestingly, these patients showed a LVGFI in the lowest group (27%) [13].

As shown in our data, low LVGFI is an independent risk factor for the worse outcome defined as death or hospitalization for heart failure. In Kaplan–Meier analysis LVEF_MRI_ also showed predictive value. We were able to show an additive value of the LVGFI in comparison to the LVEF_echo_, but to evaluate whether the LVGFI has an additive value above the LVEF_MRI_, this study only generated a hypothesis. To clarify if one of these parameters is superior to the other, studies with a larger cohort are required.

Since there was an absolute need for ICD implantation in all of these patients after surviving a cardiac arrest, there was no value of low LVGFI for selecting patients who need ICD and who do not.

There was a high occurrence of appropriate therapies in this cohort, but most of them received only one appropriate therapy. No significant difference could be shown between patients with a greater LVGFI and patients with a lower one, regarding the occurrence of multiple appropriate therapies or the performance of VT ablation. However, the focus of this study was on the occurrence of VT or VF in general, not on the frequency of its appearance.

In this study, our focus was on secondary prevention ICD patients, since there were no data available regarding the LVGFI in this cohort so far, but it should be said that neither data regarding the LVGFI in primary prevention ICD patients is available. Thus, and due to the promising data received about secondary prevention patients in this study, the LVGFI should also be evaluated in primary prevention ICD patients.

Low LVGFI was able to identify a high-risk collective among these secondary prevention ICD-patients and could be used to stratify the patients who should be followed strictly and need much more attention to modifying risk factors.

Another important finding in our study was that although lower LVGFI and lower LVEF_MRI_ were correlated with worse outcomes in Kaplan–Meier analysis, the EF measured with echocardiography did not have any additive value. This again showed the limited value of LVEF_echo_ in selecting patients with higher risk in secondary prevention populations and that the LVGFI might be a valid parameter to stratify a group among these patients which should be followed up more regularly than the whole collective.

### Limitations

This is an observational study with all the known problems of analyzing data in a retrospective manner and should be regarded as hypothesis generating data. Regarding arrhythmias, the focus of the current study was on the appearance of VT and VF in general and, therefore, recorded on the basis of a time-to-first-event approach. Medication was recorded only in the baseline characteristics. There is no information regarding change of drugs or doses during follow up. Additionally, the frequency of appropriate therapies was not analyzed. Furthermore, the presence of LGE was detected, but due to the fact that the quantification of LGE is not included in our regular CMR protocol we are not able to make a statement about its extend. Additionally, neither T1 values, ECV nor CMR based strain were available for these patients. Although the patients were followed for a long period the study population was rather small and studies with greater patient populations are needed to better define the value of LVGFI in determining the prognosis in different patient groups.

## 5. Conclusions

In summary, LVGFI was shown as an independent predictor for adverse events in secondary prevention ICD-patients and can be used to detect a high-risk group among these patients, but further studies with greater patient populations are required to evaluate the prognostic value of the LVGFI in different patient groups. Additionally, the value of LVEF_MRI_ should also be evaluated in a larger cohort especially in comparison to LVGFI, since this study generated a hypothesis, but could not proof superiority of one parameter. A head-to-head comparison with another promising parameter, such as strain analysis and LGE quantification, should be included. Regarding the promising data we could present about the LVGFI in secondary prevention ICD patients, this marker should also be evaluated in primary prevention ICD patients.

## Figures and Tables

**Table 1:** Baseline characteristics.

	Total Study Cohort (105)
Age	56 (43; 65)
Male sex	80 (76.2%)
Device	
1C	62 (59.0%)
2C	25 (23.8%)
CRT-D	7 (6.7%)
S-ICD	11 (10.5%)
LVEF ≤ 35%	25 (23.8%)
LVEF (Echo)	45.0 (35.0; 57.0)
LVEF (MRI)	41.0 (27.6; 56.5)
LVGFI	24.5 (15.1; 33.7)
LGE	68 (64.8%)
NYHA ≥ II	64 (61%)
MI	27 (25.7%)
CAD	43 (41.0%)
Cardiomyopathy	
DCM	20 (19.0%)
Other cardiomyopathy *	19 (18.1%)
Primary VF	10 (9.5%)
Long-QT-Syndrome	7 (6.7%)
Short-QT-Syndrome	1 (0.9%)
Idiopathic VT	5 (4.8%)
peripheral arterial occlusive disease (PAOD)	6 (5.7%)
Valve surgery	3 (3.1%)
AF	20 (19.0%)
Hypertension	51 (48.6%)
Diabetes	17 (16.2%)
Renal insufficiency	12 (11.4%)
TIA/stroke	5 (4.8%)
Smoking	48 (45.7%)
COPD	5 (4.8%)
ACE-inhibitors/ATI/Entresto	74 (70.5%)
Beta-Blocker	77 (73.3%)
Ca-Antagonists	10 (9.5%)
Diuretics	42 (40.0%)
Spironolactone	45 (42.9%)
ASS	39 (37.1%)
Macumar/NOAKs	22 (21.0%)
P2Y12	13 (12.4%)
Amiodaron/Sotalol	5 (4.8%)
Digitalis	5 (4.8%)
Statin	41 (39.0%)
Ivabradin	1 (1.0%)
Monomorph VT	46 (43.8%)
Polymorph VT	5 (4.8%)
Ventricular Flutter	1 (1.0%)
Ventricular Fibrillation	48 (45.6%)
Asystole	5 (4.8%)

* Other cardiomyopthy includes HCM or HOCM, ARVD, Tako Tsubo cardiomyopathy, residuals after myocarditis and non-compaction cardiomyopathy. 1C: single-chamber ICD; 2C: dual-chamber ICD; CRT-D: implantable cardiac resynchronization therapy defibrillators; S-ICD: subcutaneous implantable cardioverter defibrillators; LGE: late gadolinium enhance-ment; MI: myocardial infarction; CAD: coronary artery disease; VF: ventricular fibrillation; AF: atrial fibrillation; TIA: transient ischaemic attack; COPD: chronic obstructive pulmonary disease; ACE: Angiotensin-converting enzyme; AT1: Angioten-sin 1-receptor antagonists; ASS: acetylsalicylic acid; NOAKs: new oral anticoagulants; P2Y12: Clopidogrel/Prasugrel/Ticagrelor; LVEF: left ventricular (LV) ejection fraction; LVGFI: LV global function index; VT: ventricular tachycardia.

**Table 2:** Baseline characteristics of patients according to their LGVFI.

	LVGFI ≤ 13.5% (19)	LVGFI > 13.5% (86)	*p*
Age	58 (47; 68)	55 (40.8; 64.0)	0.316
Male sex	15 (78.9%)	65 (75.6%)	0.756
Device			0.996
1C	11 (57.8%)	51 (59.3%)	
2C	4 (21.1%)	21 (24.4%)	
CRT-D	4 (21.1%)	3 (3.5%)	
S-ICD	0 (0%)	11 (12.8%)	
**LVEF** **≤** **35%**	**13 (68.4%)**	**12 (14.0%)**	**<0.001**
**LVEF (Echo)**	**23.0 (19.0; 41.0)**	**48.0 (39.8; 58.3)**	**<0.001**
**LVEF (MRI)**	**16.0 (14.0; 21.0)**	**46.0 (36.6; 60.3)**	**<0.001**
**LVGFI**	**10.2 (8.1; 12.4)**	**28.3 (21.0; 36.5)**	**<0.001**
LGE	13 (68.4%)	55 (80.1%)	0.685
**NYHA** **≥** **II**	**16 (84.2%)**	**48 (55.8%)**	**0.022**
MI	8 (42.1%)	19 (22.1%)	0.099
CAD	11 (57.9%)	32 (37.2%)	0.072
**Cardiomyopathy**			**0.025**
**DCM**	**7 (36.8%)**	**13 (15.1%)**	
Other cardiomyopathy *	**1 (5.2%)**	**18 (20.9%)**	
Primary VF	0 (0%)	10 (11.6%)	0.120
Long-QT-Syndrome	0 (0%)	7 (8.1%)	0.200
Short-QT-Syndrome	0 (0%)	1 (1.3%)	0.638
Idiopathic VT	0 (0%)	5 (5.8%)	0.284
PAOD	1 (5.3%)	5 (5.8%)	0.926
Valve surgery	0 (0%)	3 (3.5%)	0.411
AF	3 (15.8%)	17 (19.8%)	0.691
Hypertension	13 (68.4%)	38 (44.2%)	0.057
**Diabetes**	**6 (31.6%)**	**11 (12.8%)**	**0.045**
Renal insufficiency	4 (21.1%)	8 (9.3%)	0.147
TIA/stroke	0 (0%)	5 (5.8%)	0.284
Smoking	9 (47.4%)	39 (45.3%)	0.934
COPD	0 (0%)	5 (5.8%)	0.284
ACE-inhibitors/ATI/Entresto	14 (73.7%)	60 (69.8%)	0.736
Beta-Blocker	16 (84.2%)	61 (70.9%)	0.238
Ca-Antagonists	2 (10.5%)	8 (9.3%)	0.870
Diuretics	16 (84.2%)	26 (30.2%)	<0.001
Spironolactone	14 (73.7%)	31 (36.0%)	0.003
ASS	9 (47.4%)	30 (34.9%)	0.310
Macumar/NOAKs	6 (31.6%)	16 (18.6%)	0.211
P2Y12	4 (21.1%)	9 (10.5%)	0.207
Amiodaron/Sotalol	3 (15.8%)	2 (2.3%)	0.013
Digitalis	2 (10.5%)	3 (3.5%)	0.194
Statin	7 (36.8%)	34 (39.5%)	0.800
Ivabradin	0 (0%)	1 (1.2%)	0.630
Monomorph VT	11 (57.9%)	35 (40.7%)	0.174
Polymorph VT	0 (0%)	5 (5.8%)	0.284
Ventricular Flutter	0 (0%)	1 (1.2%)	0.638
Ventricular Fibrillation	6 (31.6%)	42 (48.8%)	0.174
Asystole	2 (10.5%)	3 (3.5%)	0.194

* Other cardiomyopthy includes HCM or HOCM, ARVD, Tako Tsubo cardiomyopathy, residuals after myocarditis and non-compaction cardiomyopathy. The bold format show the ones with *p* < 0.05.

**Table 3:** Cox-regression analysis.

	Hazard-Ratio	*p*
LVGFI	0.938 (0.887–0.991)	0.023
Older age at implantation	1.060 (1.014–1.107)	0.010
LVEF (MRI)	1.021 (0.926–1.124)	0.673
LVEF (Echo)	0.984 (0.930–1.041)	0.876
NYHA ≥ II	0.488 (0.129–1.841)	0.483
presence of diabetes	0.714 (0.194–2.622)	0.384
diuretic usage	1.476 (0.426–5.119)	0.331
Spironolactone usage	0.513 (0.183–1.438)	0.271
Amiodaron or Sotalol usage	4.081 (0.518–32.149)	0.150
Female sex	0.414 (0.150–1.139)	0.087
